# Neoadjuvant Immune Checkpoint Inhibitors in hepatocellular carcinoma: a meta-analysis and systematic review

**DOI:** 10.3389/fimmu.2024.1352873

**Published:** 2024-02-19

**Authors:** Chunhong Tian, Yifan Yu, Yuqing Wang, Lunwei Yang, Ying Tang, Chengyang Yu, Gaofei Feng, Dayong Zheng, Xiongwen Wang

**Affiliations:** ^1^ The First Clinical Medical School, Guangzhou University of Chinese Medicine, Guangzhou, China; ^2^ Institute of Tumor, Guangzhou University of Chinese Medicine, Guangzhou, China; ^3^ Science and Technology Innovation Center, Guangzhou University of Chinese Medicine, Guangzhou, China; ^4^ Department of Oncology, Shenzhen Hospital, Beijing University of Chinese Medicine, Shenzhen, China; ^5^ Department of Hepatology, TCM-Integrated Hospital of Southern Medical University, Guangzhou, China; ^6^ Department of Hepatopancreatobiliary, Cancer Center, Southern Medical University, Guangzhou, China; ^7^ Department of Oncology, Guangzhou University of Chinese Medicine, Guangzhou, China; ^8^ Beibei District Traditional Chinese Medicine Hospital (Chongqing Hospital, The First Affiliated Hospital of Guangzhou University of Chinese Medicine), Chongqing, China; ^9^ Department of Oncology, The First Affiliated Hospital of Guangzhou University of Chinese Medicine, Guangzhou University of Chinese Medicine, Guangzhou, China

**Keywords:** neoadjuvant, immunotherapy, hepatocellular carcinoma (HCC), resectable, systematic review, meta-analysis

## Abstract

**Background:**

Neoadjuvant immunotherapy has demonstrated beneficial outcomes in various cancer types; however, standardized protocols for neoadjuvant immunotherapy in hepatocellular carcinoma (HCC) are currently lacking. This systematic review and meta-analysis aims to investigate the reliability of neoadjuvant immunotherapy’s efficacy and safety in the context of HCC.

**Methods:**

A systematic search was conducted across PubMed (MEDLINE), EMBASE, the Web of Science, the Cochrane Library, and conference proceedings to identify clinical trials involving resectable HCC and neoadjuvant immunotherapy. Single-arm meta-analyses were employed to compute odds ratios and 95% confidence intervals (CIs). Heterogeneity analysis, data quality assessment, and subgroup analyses based on the type of immunotherapy drugs and combination therapies were performed. This meta-analysis is registered in PROSPERO (identifier CRD42023474276).

**Results:**

This meta-analysis included 255 patients from 11 studies. Among resectable HCC patients, neoadjuvant immunotherapy exhibited an overall major pathological response (MPR) rate of 0.47 (95% CI 0.31-0.70) and a pathological complete response (pCR) rate of 0.22 (95% CI 0.14-0.36). The overall objective response rate (ORR) was 0.37 (95% CI 0.20-0.69), with a grade 3-4 treatment-related adverse event (TRAE) incidence rate of 0.35 (95% CI 0.24-0.51). Furthermore, the combined surgical resection rate was 3.08 (95% CI 1.66-5.72). Subgroup analysis shows no significant differences in the efficacy and safety of different single-agent immunotherapies; the efficacy of dual ICIs (Immune Checkpoint Inhibitors) combination therapy is superior to targeted combined immunotherapy and monotherapy, while the reverse is observed in terms of safety.

**Discussion:**

Neoadjuvant immunotherapy presents beneficial outcomes in the treatment of resectable HCC. However, large-scale, high-quality experiments are warranted in the future to provide robust data support.

## Introduction

Hepatocellular Carcinoma (HCC) is one of the most prevalent malignant tumors globally and ranks as the third leading cause of cancer-related deaths worldwide, with a relative 5-year survival rate of only about 18% (1, 2). Surgical resection stands as the primary treatment choice for HCC patients, particularly for those with a single tumor and preserved normal liver function (3). However, even among resectable HCC patients, long-term survival outcomes post-surgery are less than ideal. Up to 80% face the risk of recurrence within 5 years after surgery, with many diagnosed patients already in the late stages of the disease, presenting visible vascular invasion and multifocal intrahepatic metastases (4, 5). Neoadjuvant therapy, incorporating various treatment modalities such as drugs and radiation, aims to improve overall treatment efficacy, reduce recurrence risk, and shrink lesions, transforming initially unresectable diseases into manageable therapeutic strategies, offering multiple potential advantages for HCC treatment (6, 7). Despite the absence of established consensus or standardized protocols for neoadjuvant therapy in HCC patients, evaluations have been carried out regarding potential neoadjuvant options like transarterial chemoembolization and transarterial radioembolization. However, the results have not yielded satisfactory survival benefits and are not considered optimal choices for improving overall survival in patients (8, 9). In recent years, global guidelines for HCC encompass five monotherapy molecular treatment approaches. Among these, immune checkpoint inhibitors, primarily PD-1/PD-L1 inhibitors, have opened up new avenues for neoadjuvant immunotherapy in HCC patients (10–12).The PD-1/PD-L1 pathway serves as a crucial mediator of local immune suppression within the tumor microenvironment (TME). By blocking negative regulatory mechanisms, it reactivates T-cell immune responses against tumors, aiming for antitumor effects (13, 14). The use of anti-PD-(L)1 immunotherapy in the neoadjuvant (preoperative) setting presents a potential therapeutic option for achieving “resectable cure” in tumors (15). Furthermore, combination therapies involving immune checkpoint inhibitors and anti-angiogenic agents exhibit enhanced survival benefits for HCC patients compared to standalone immune checkpoint inhibitor therapy (16). However, clinical studies supporting neoadjuvant immunotherapy for HCC are relatively scarce, and its treatment efficacy and safety remain unknown. There is a lack of standardized protocols for neoadjuvant immunotherapy in clinical practice (7).

Hence, based on the current clinical research findings, this article aims to discuss the effectiveness and safety of neoadjuvant immunotherapy in resectable HCC patients. The objective is to provide an objective and comprehensive evaluation of existing studies, offering more reliable and stable references. This discussion aims to support the feasibility assessment of neoadjuvant immunotherapy in HCC treatment and provide additional evidence-based medicine for clinical treatment.

## Materials and methods

This study followed the PRISMA reporting guidelines for systematic reviews and meta-analysis (17). This meta-analysis has been registered in PROSPERO with the identifier CRD42023474276.

### Search strategy

We performed computer-based searches in PubMed (MEDLINE), EMBASE, the Web of Science, and the Cochrane library. Additionally, manual searches were conducted to include references from the literature. To ensure comprehensive data collection, we reviewed abstracts and reports from the American Society of Clinical Oncology (ASCO) and the European Society for Medical Oncology (ESMO) conferences until October 15, 2023. Initially, we determined relevant subject headings and free-text terms through a MESH query and used Boolean operators for combination search strategies. Search terms comprised medical subject headings including “Hepatocellular Carcinoma,” “neoadjuvant therapy,” “immune checkpoint inhibitors,” and related variations. The search duration for all databases encompassed records from their inception to October 15, 2023.

### Study selection

We developed the inclusion and exclusion criteria based on the patient, intervention, comparison outcomes (PICOs) principles of evidence-based medicine (EBM). The following criteria were used to select studies for inclusion: (1) Patients with resectable hepatocellular carcinoma. (2) Administration of immune checkpoint inhibitors as neoadjuvant therapy. (3) Reports containing at least one of the following primary outcomes: Major Pathological Response (MPR), Pathological Complete Response (pCR), Grade 3-4 Treatment-Related Adverse Event Rate (Grade 3-4 TRAEs), Objective Response Rate (ORR), Resection Rate. Publications that met one of the following criteria were excluded: (1) Patients with unresectable primary or metastatic diseases. (2) Patients with a history of prior immunotherapy or other systemic treatments. (3) Studies with outcomes not directly related to our specified key outcomes. (4) Duplicate publications. (5) Reviews, meta-analyses, case reports, or case series.

### Data extraction

Two researchers independently conducted the identification and extraction of potentially eligible articles. Any discrepancies were resolved by involving a third reviewer. Subsequently, the identified articles were retrieved, and a comprehensive analysis of their full texts was performed. For each study, a range of data was meticulously recorded, encompassing details such as the first author, publication year, clinical trial status, NCT number, intervention model, blinding, study type, study phase, geographical location, article type, specific immune checkpoint inhibitor (ICI) drugs, sample size, pathological complete response (pCR), major pathological response (MPR), Grade 3-4 TRAEs, objective response rate (ORR), and resection rate. In cases where this data was unavailable, calculations were made based on the information provided within the articles.

### Quality assessment and risk of bias

We utilized the Methodological Index for Non-Randomized Studies (MINORS) quality assessment tool to evaluate the quality of non-randomized studies. The assessment covered specific criteria including the clarity of the study’s objectives, consistency in patient inclusion, collection of anticipated data, appropriateness of endpoints reflecting the study’s objectives, objectivity in endpoint evaluation, adequacy of follow-up, dropout rates below 5%, and the consideration of sample size estimation. Each criterion was scored on a scale from 0 to 2: 0 indicated unreported, 1 reported but inadequately, and 2 reported and fully detailed, with an ideal score of 16. The review authors’ judgments of the risk of bias for each item are presented in **Supplementary (Figure S)**.

### Data analysis

In single-arm trials lacking a control group, the uncontrolled binary data need conversion. The transformation formula used is:*p*=ln(odds)=ln[*X*/(*n−X*)], Here, *n* represents the total number of included patients, *X* indicates the number of events, *p* signifies the occurrence rate, and SE(*p*)=SE[ln(odds)] = $\sqrt{1/X+1/(n-X}$ , where SE(*p*) stands for the standard error; Subsequently, the odd ratio (OR) and its 95% confidence interval (CI), Actual occurrence rates(*p*_f_) and their 95% CI are obtained by using the formula *p*_f_=OR/(1+OR),The lower limit of the 95% CI (LL) is calculated as(LL)=LL_OR_/(1+LL_OR_) and the upper limit of the 95% CI(UL)=UL_OR_/(1+UL_OR_).Heterogeneity among the included studies was analyzed using the *χ* ^2^ test and *I* ^2^ test, if *P*<0.1, *I* ^2^ ≥50%,it indicates statistical heterogeneity among the studies, thus requiring the utilization of a random-effects model for the combined analysis. If not, a fixed-effects model was employed for the combined analysis.

## Results

### Literature screening results and basic information of included studies

**Figure 1:**
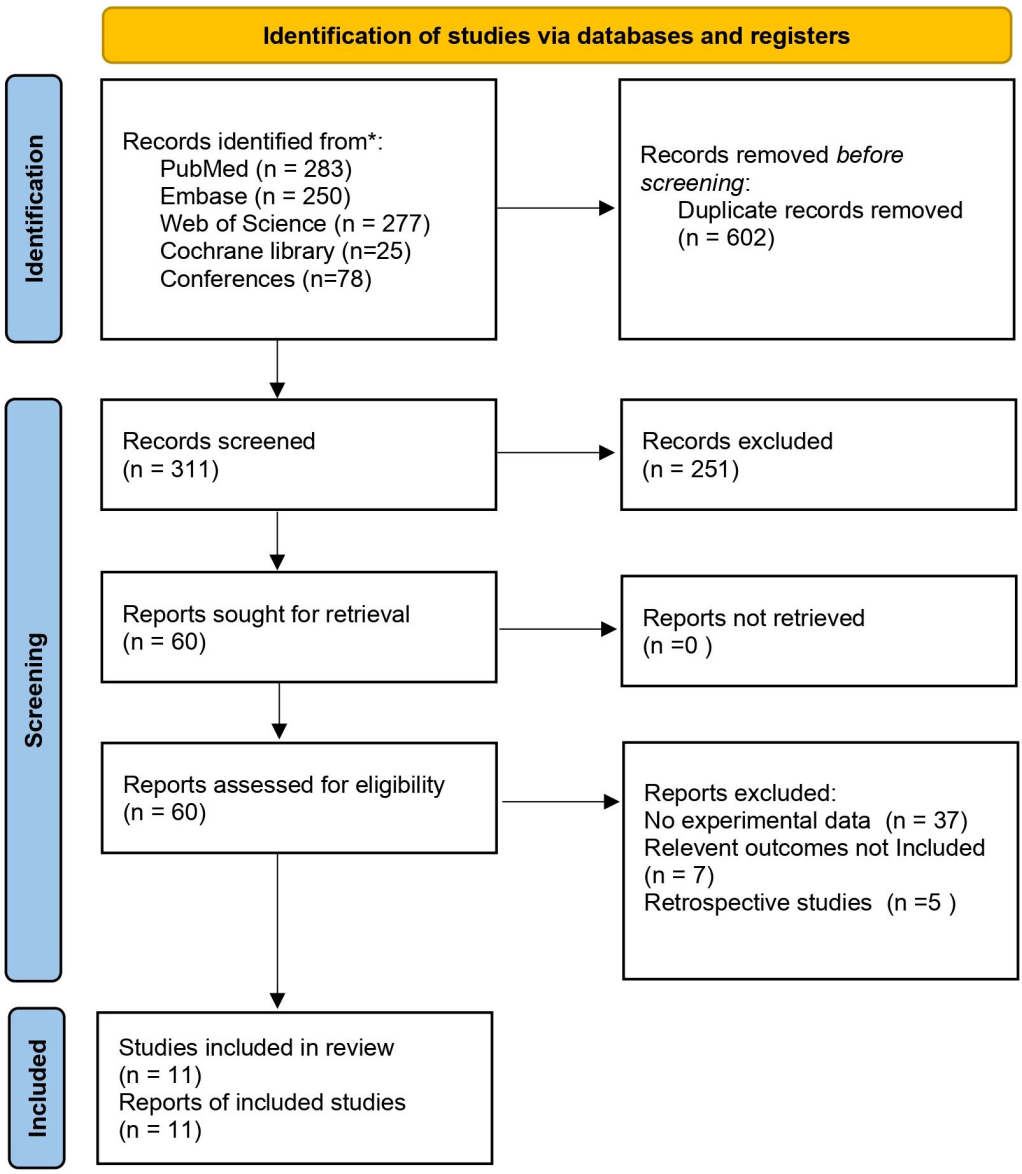
Flowchart of study selection.

A total of 913 relevant articles were initially identified. After removing duplicate articles and reviewing titles, abstracts, and full texts, a final selection of 11 articles (18–28), comprising 255 patients, was included. The flowchart of the literature screening process is illustrated in **Figure 1**, and the basic information of the included studies is provided in **Table 1**.

**Table 1:** Characteristics of neoadjuvant immunotherapy studies in patients with hepatocellular carcinoma.

Source (Author/Year)	Trial Indentifier	Region	Sample Size	Study Phase	Intervention Model	Masking	Study Type	Randomization Method	Article Type	Neoadjuvant Immuotherapy	ICIs Post-Surgery	pCR	MPR	Grade3–4 TRAEs	ORR	Resection rate
Shi, Y.H. et al., (20)	NCT03867370	China	18	1b/2	Sequential Assignment	Open Label	Clinical Trial	Randomized	Conference abstract	Toripalimab (n = 14) or Toripalimab+ Lenvatinib (n = 4)	Yes	6.3%(1/16)	NA	16.7%(3/18)	NA	NA
Su, Y. et al., (18)	NCT03510871	China	29	2	Single Group Assignment	Open Label	Clinical Trial	N/A	Conference abstract	Nivolumab+ ipilimumab	N/A	NA	33.3%(5/15)	41.4%(12/29)	NA	51.7%(15/29)
Ho, W.J. et al., (19)	NCT03299946	USA	15	1	Single Group Assignment	Open Label	Clinical Trial	N/A	Full text	Nivolumab+ Cabozantinib	N/A	8.3%(1/12)	33.3%(4/12)	13.3%(2/15)	NA	80%(12/14)
Marron,T.U. et al., (25)	NCT03916627	USA	21	2	Single Group Assignment	Open Label	Clinical Trial	N/A	Full text	Cemiplimab	Yes	15%(3/20)	20%(4/20)	10%(2/21)	15%(3/20)	95.2%(20/21)
Xia, Y. et al., (21)	NCT04297202	China	20	2	Single Group Assignment	Open Label	Clinical Trial	N/A	Full text	Camrelizumab+ Apatinib	Yes	5.9%(1/17)	17.6%(3/17)	16.7%(3/18)	16.7%(3/18)	94.4%(17/18)
Kaseb, A.O. et al., (22)	NCT03222076	USA	30	2	Parallel Assignment	Open Label	Clinical Trial	Randomized	Full text	Nivolumab (n = 13) or Nivolumab +Ipilimumab (n = 14)	Yes	25%(5/20)	30%(6/20)	33.3%(9/27)	NA	74.1%(20/27)
Chen, S. et al., (23)	NCT04615143	China	11	2	Sequential Assignment	Open Label	Clinical Trial	Non-Randomized	Conference abstract	Tislelizumab	Yes	9.1%(1/11)	NA	NA	18.2% (2/11)	NA
Bai, X. et al., (24)	NCT04930315	China	32	2	Parallel Assignment	Open Label	Clinical Trial	Randomized	Conference abstract	Camrelizumab+ Apatinib (n = 16)	Yes	9.1%(1/11)	27.3%(3/11)	NA	NA	NA
Song,T.Q. et al., (27)	NCT04834986	China	24	2	Single Group Assignment	Open Label	Clinical Trial	N/A	Conference abstract	Tislelizumab +Lenvatinib	Yes	17.6%(3/17)	35.3%(6/17)	NA	54.2%(13/24)	70.8%(17/24)
D’Alessio,A. et al., (28)	NCT03682276	UK	25	1b	Single Group Assignment	Open Label	Clinical Trial	N/A	Conference abstract	Nivolumab+ Ipilimumab	N/A	38%(6/16)	56%(9/16)	NA	29%(6/21)	84%(21/25)
Sun,H.C.et al., (26)	NCT04843943	China	30	2	Single Group Assignment	Open Label	Clinical Trial	N/A	Conference abstract	Sintilimab+ Bevacizumab	N/A	N/A	N/A	23.3%(7/30)	26.7%(8/30)	56.7%(17/30)

N/A, not applicable; ICIs, immune checkpoint inhibitors.

### Efficacy of neoadjuvant immune checkpoint inhibitors

**Figure 2:**
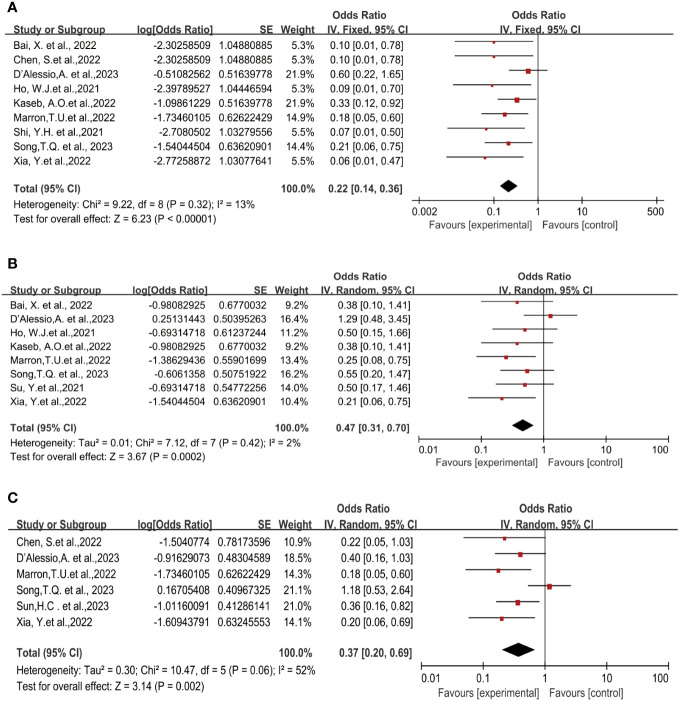
Forest plot of the efficacy of neoadjuvant immune checkpoint inhibitors in resectable hepatocellular carcinoma. **(A)** pCR, **(B)** MPR, **(C)** ORR. pCR, pathological complete response; MPR, major pathological response; ORR, Overall Response Rate.

Nine studies reported the pathological complete response (pCR) rates ranging from 5.9% to 38% (19–25, 27, 28). No statistical heterogeneity was observed among the studies (*P*=0.32, *I^2 = ^* 13%), hence a fixed-effect model was employed for meta-analysis. The pooled results of the included trials indicated a statistically significant benefit with the use of neoadjuvant Immune Checkpoint Inhibitors (ICI) in terms of pCR rates [OR=0.22, 95%CI (0.14, 0.36), *P*<0.00001], as illustrated in **Figure 2A**.

Eight studies reported the major pathological response (MPR) rates (18, 19, 21–25, 28), ranging from 17.6% to 56%. In terms of MPR, individual odds ratios (ORs) from each eligible study supported the use of neoadjuvant ICI (individual OR<1.0). No statistical heterogeneity was observed among the studies (*P*=0.42, *I^2 = ^* 2%), and a fixed-effect model was applied for meta-analysis. The results demonstrated that neoadjuvant ICI showed significant benefits, with a statistically significant difference in MPR rates [OR=0.47, 95%CI (0.31, 0.70), *P*=0.0002], as shown in **Figure 2B**.

Six studies reported the Objective Response Rate (ORR) (21, 23, 25–28), ranging from 15% to 52.2%. Significant heterogeneity existed among the studies (*P*=0.06, *I^2 = ^* 52%), hence a random-effects model was utilized for meta-analysis. The results indicated a significant effect on Objective Response Rate (ORR) with the use of neoadjuvant ICI, showing a statistically significant difference in ORR rates [OR=0.37, 95%CI (0.20, 0.69), *P*<0.002], as depicted in **Figure 2C**.

### Safety of neoadjuvant immune checkpoint inhibitors

**Figure 3:**
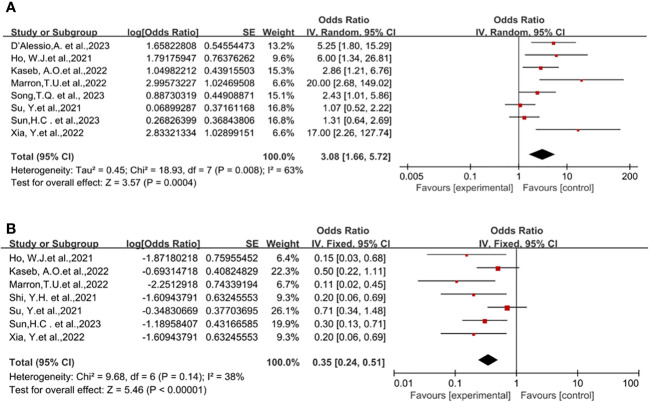
Forest plot of the safety of neoadjuvant immune checkpoint inhibitors in resectable hepatocellular carcinoma. **(A)** Resection Rate, **(B)** Grade 3–4 TRAEs. TRAEs, treatment-related adverse events.

Eight studies reported on surgical resection (18, 19, 21–23, 25–28), displaying statistical heterogeneity among the studies (P=0.008, *I^2 = ^* 63%). A random-effects model was used for meta-analysis (**Figure 3A**). The pooled Odds Ratio (OR) for resection rate was 3.08 (95% CI, 1.66-5.72) with a p-value of 0.0004.

The incidence of Treatment-Related Adverse Events (TRAEs) is commonly associated with the safety of neoadjuvant ICI in clinical trial studies. No patient deaths due to TRAEs were reported across all trials. Preoperative grade 3-4 TRAEs included pneumonia, drug-induced hepatitis, itching, maculopapular rash, severe muscular weakness, elevated lipase, pancreatitis, immune-mediated diarrhea, and colitis, among others. A pooled analysis of seven studies indicated an OR of 0.35 (95% CI, 0.24-0.51) (18–22, 25–27), displaying no statistical heterogeneity among the studies (*P*=0.14, *I^2 = ^* 38%), and a fixed-effect model was employed for meta-analysis. The results demonstrated a benefit in terms of safety for neoadjuvant ICI (*P*<0.00001), **Figure 3B**.

### Subgroup analysis

Through subgroup analysis, we examined clinical outcomes such as pCR, MPR, ORR, TRAEs, and surgical resection rates, studying whether different treatment regimens have differences in efficacy and safety, and whether they exert varying influences on clinical outcomes.

**Figure 4:**
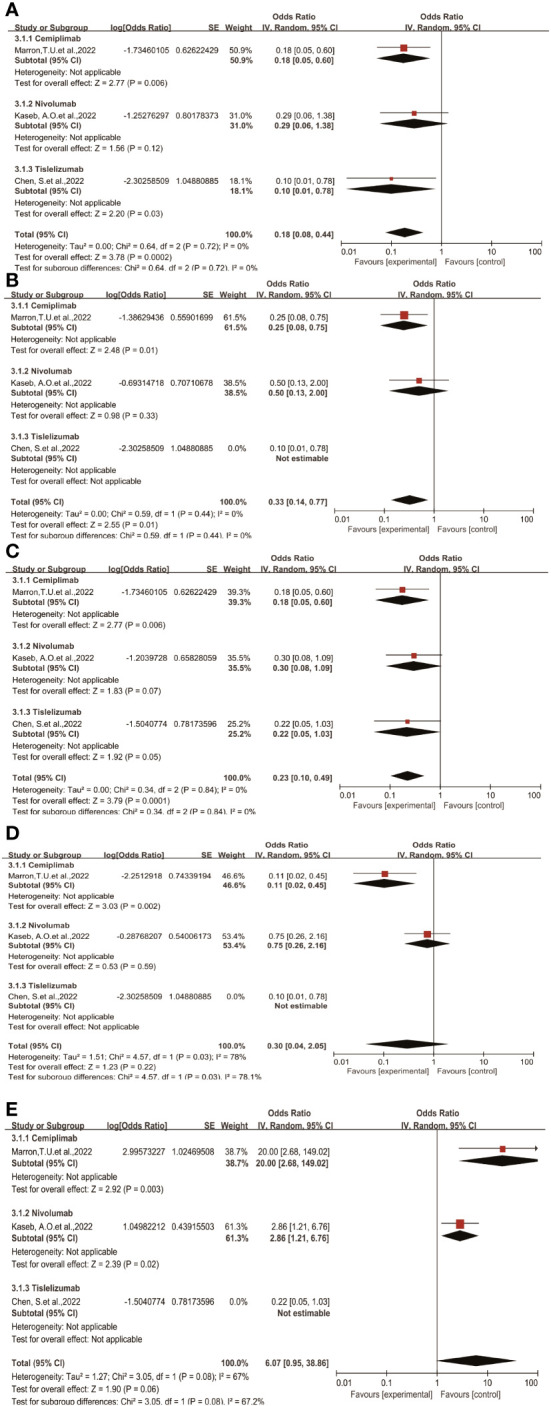
Subgroup analyses of immune checkpoint inhibitor drug types for **(A)** pCR, **(B)** MPR, **(C)** ORR, **(D)** Grade3−4 TRAEs and **(E)** Resection Rate.pCR, pathological complete response; MPR, major pathological response; ORR, Overall Response Rate; TRAEs, treatment-related adverse events.

The subgroup analysis results indicate no significant differences among the three types of immune checkpoint inhibitors (ICIs) in terms of single-drug therapy (**Figures 4A–C**). However, the odds ratio (OR) of adverse events associated with nivolumab as a monotherapy (**Figure 4D**) was higher than that of Cemiplimab, showing a significant inter-group difference statistically. On the other hand, the OR of Cemiplimab as a monotherapy for the rate of excision (**Figure 4E**) was higher than that of nivolumab, indicating a difference between groups but without statistical significance.

**Figure 5:**
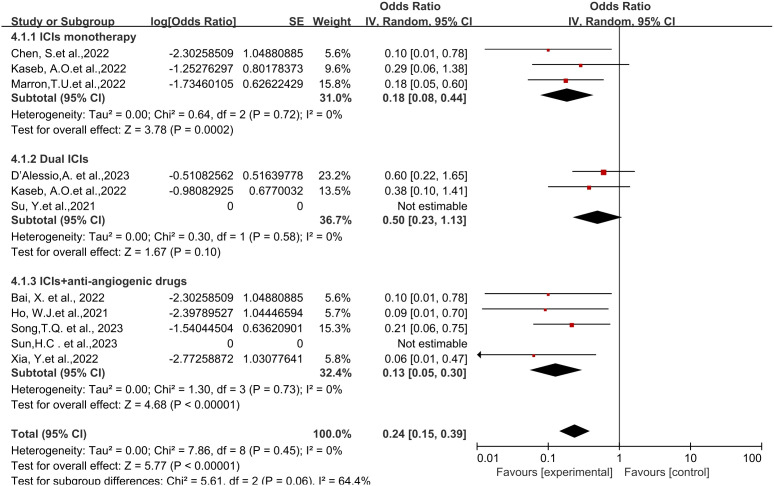
Subgroup analyses based on neoadjuvant immune checkpoint inhibitor combinations for pCR. pCR, pathological complete response.

**Figure 6:**
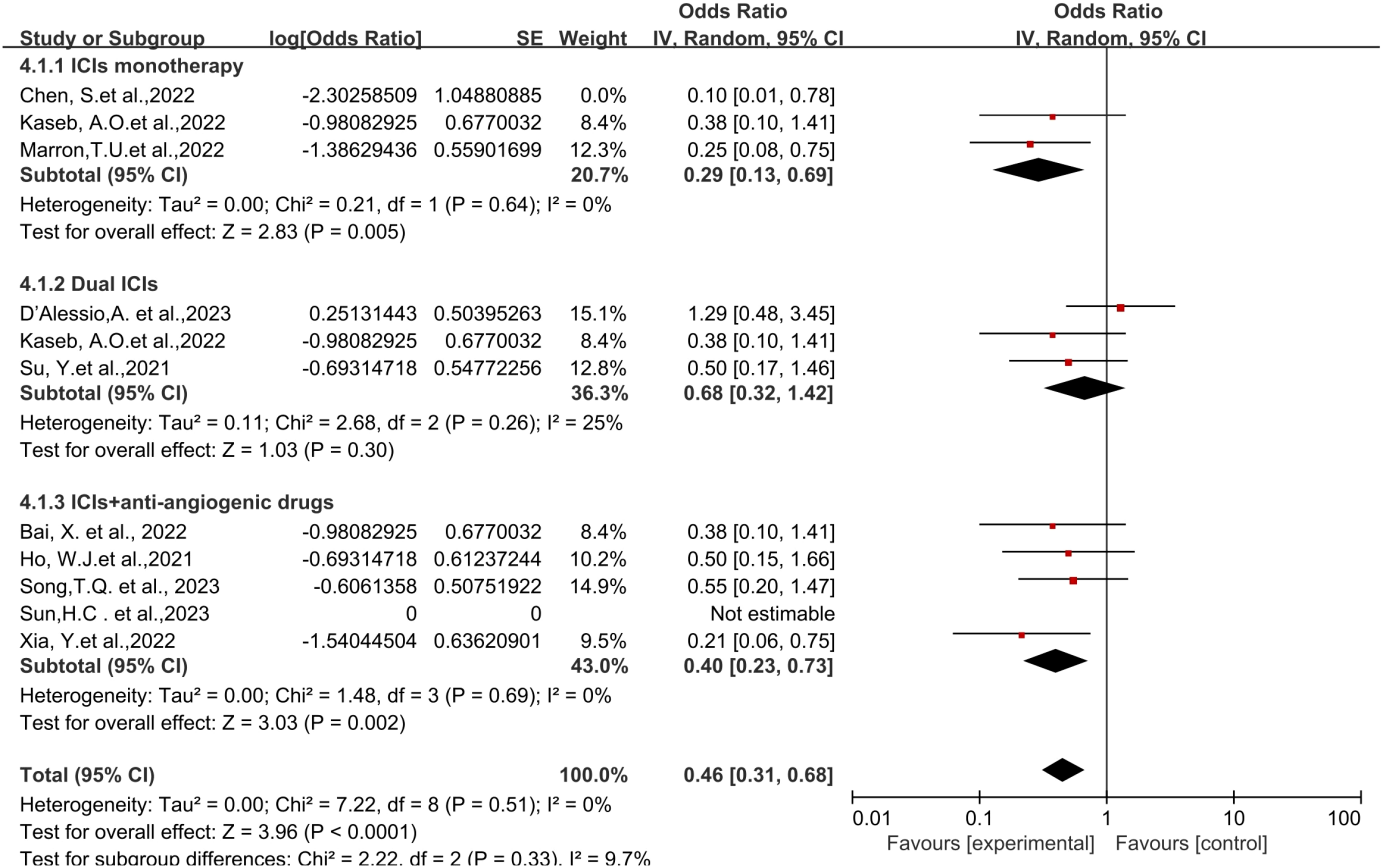
Subgroup analyses based on neoadjuvant immune checkpoint inhibitor combinations for MPR. MPR, major pathological response.

**Figure 7:**
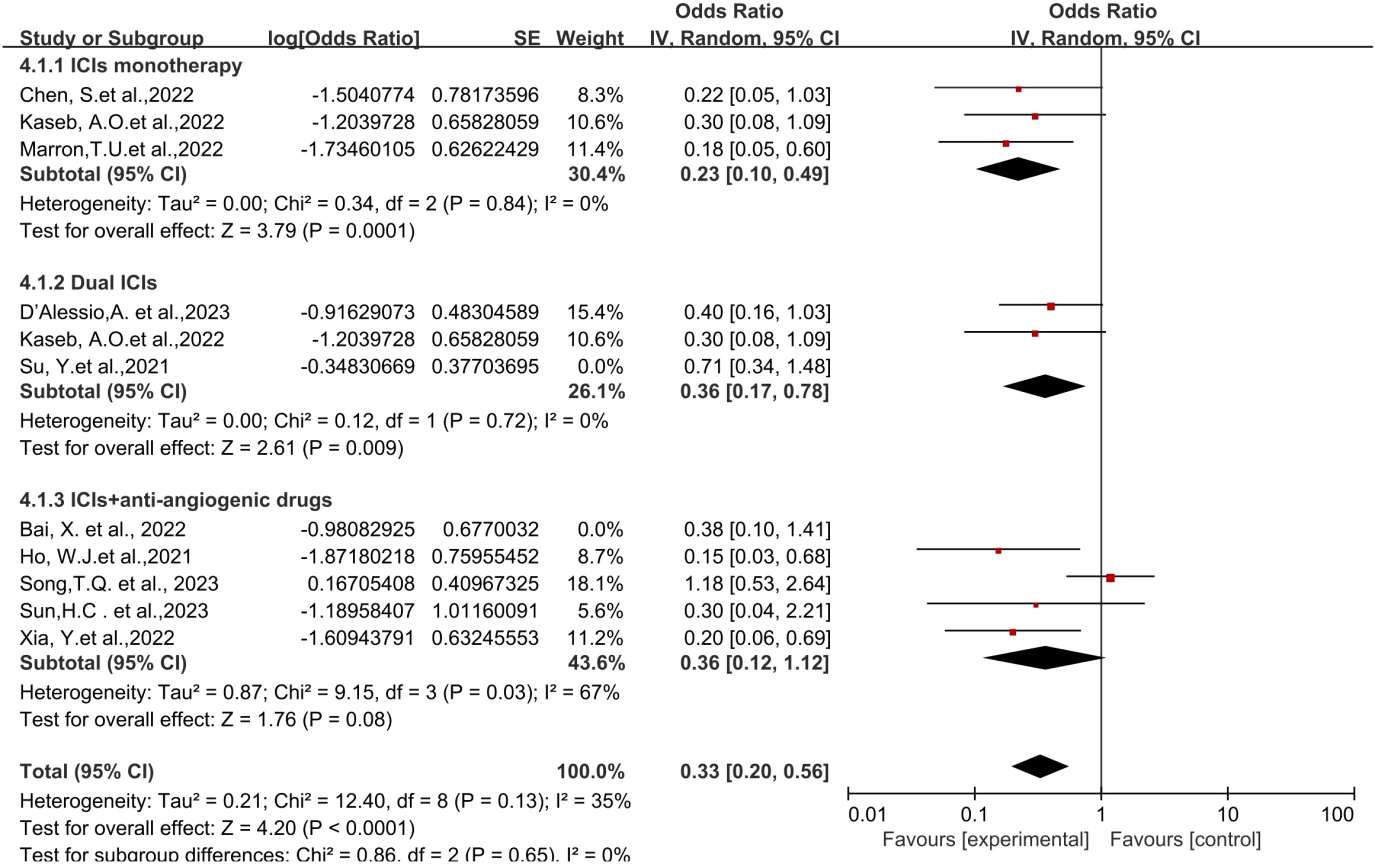
Subgroup analyses based on neoadjuvant immune checkpoint inhibitor combinations for ORR. ORR, Overall Response Rate.

**Figure 8:**
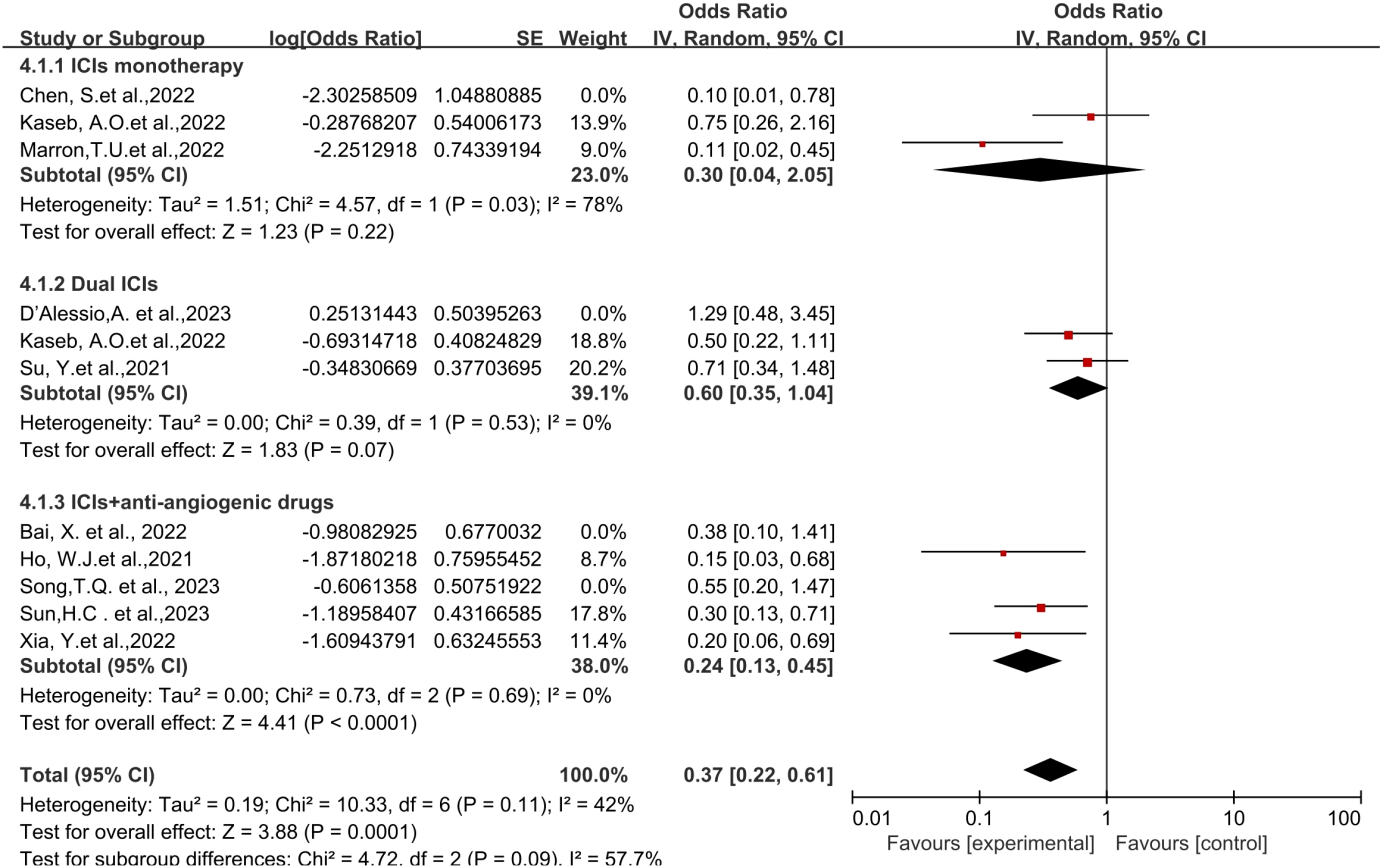
Subgroup analyses based on neoadjuvant immune checkpoint inhibitor combinations for Grade3−4 TRAEs. TRAEs, treatment-related adverse events.

**Figure 9:**
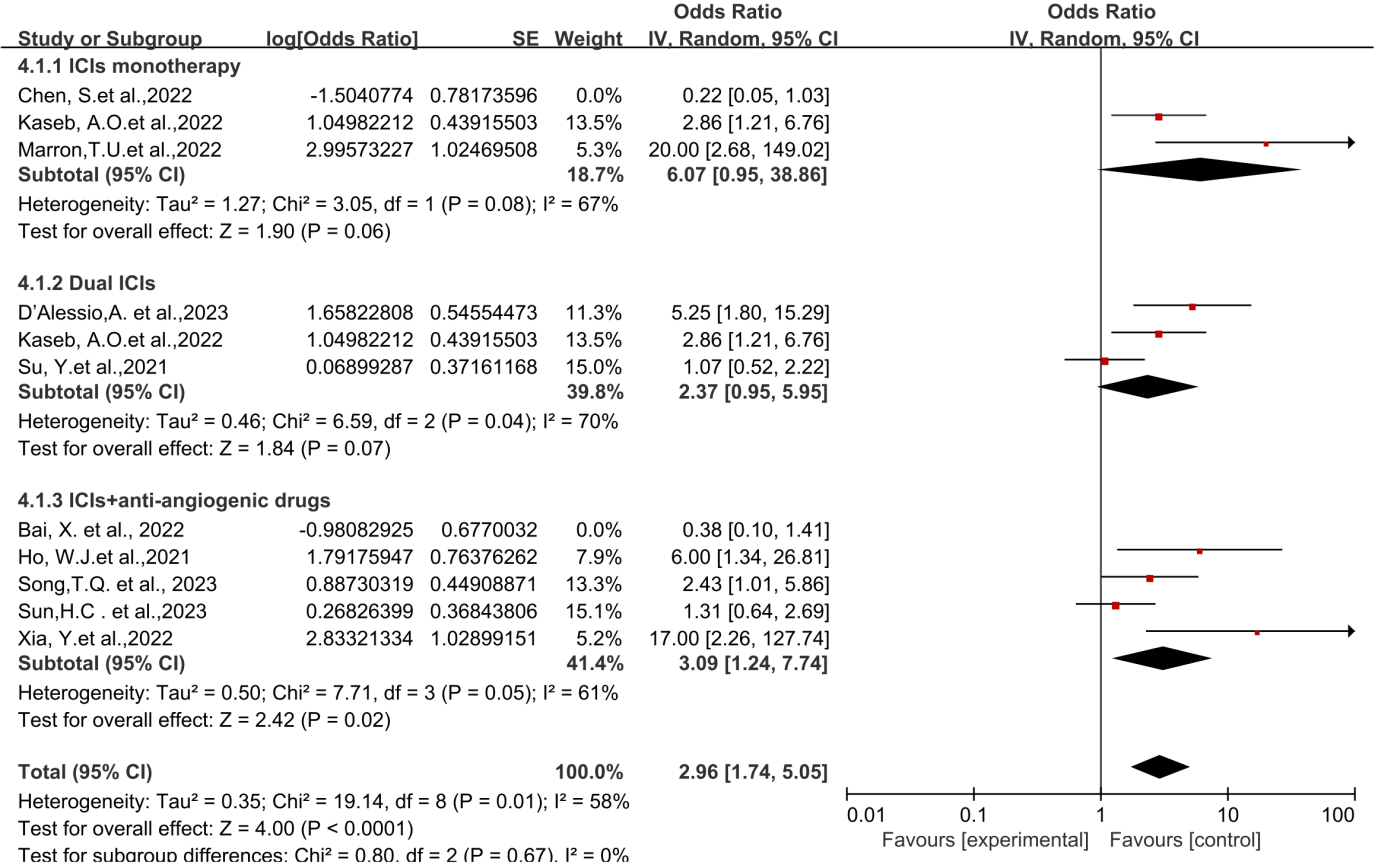
Subgroup analyses based on neoadjuvant immune checkpoint inhibitor combinations for Resection Rate.

Regarding different immune combination therapies, the combined OR for pathological complete response (pCR) with dual ICI therapy (**Figure 5**) was higher than that with single-drug therapy, while the combined OR of single-drug therapy was higher than that of immunotherapy combined with targeted therapy, showing differences between groups and statistical significance. The statistical results for major pathological response (MPR) (**Figure 6**) and objective response rate (ORR) (**Figure 7**) showed no differences among the groups. The combined OR for the occurrence of adverse events associated with dual ICI therapy (**Figure 8**) was higher than that of single-drug therapy, and the combined OR of single-drug therapy was higher than that of immunotherapy combined with targeted therapy, with significant inter-group differences and statistical significance. There were no differences observed among the groups in terms of surgical excision rates (**Figure 9**).

## Discussion

Overall, this study’s results demonstrate the advantages of neoadjuvant immunotherapy in resectable HCC. First, regarding efficacy, the meta-analysis showed an overall odds ratio (OR) of 0.37 (95% CI 0.20-0.69) for ORR, with the maximum ORR reported at 54.2% (27). The summarized ORs for pCR and MPR were 0.22 (95% CI 0.14-0.36) and (95% CI 0.31-0.70), respectively, with the maximum pCR and MPR reported at 38% and 56% (28), respectively. These results align closely with neoadjuvant immunotherapy outcomes in other tumor types (29). However, considering the ongoing nature of most studies, data regarding post-tumor resection patient survival are limited. Only four articles provided statistical data, among which Kaseb, A.O. et al. (22) reported nivolumab’s PFS as 9.4 months (95% CI 1.47–not estimable [NE]), and nivolumab plus ipilimumab’s PFS as 19.53 months (2.33–NE). Other studies (21, 26) reported EFS and RFS data with a median EFS of 13.8 months (95% CI 10.3-17.3) and a 1-year RFS of 53.85% (95% CI: 24.77%-75.99%). However, due to insufficient follow-up, none of the studies reported OS data. In other tumors, the significant correlation between pathological response and survival has been validated (30). This study also conducted statistical analysis, as Ho, W. J et al. (19) found a correlation between achieving MPR (Major Pathological Response) and long-term DFS (Disease-Free Survival). So far, all patients have had a DFS interval exceeding 230 days. Kaseb, A. O. and colleagues (22) similarly reported a significant difference in recurrence-free survival based on the presence or absence of a significant pathological response (p=0.049), despite a smaller sample size. Six patients who experienced a significant pathological response did not experience recurrence at a median follow-up of 26.8 months, whereas among the 14 patients who did not show a significant pathological response, seven experienced recurrence. Xia, Y et al. (21), on the other hand, found a higher RFS (Recurrence-Free Survival) in MPR/pCR (Pathological Complete Response) patients compared to non-MPR patients, although it did not reach statistical significance, possibly due to the small sample size.

Post-hepatectomy recurrence is a common cause of disease progression in HCC (31). Early recurrence is often the result of occult intrahepatic metastasis, closely associated with tumor burden, accounting for approximately 70% of all recurrence cases. Studies indicate that early recurrence significantly decreases overall survival and disease-free survival rates (32, 33). Neoadjuvant immunotherapy is believed to induce long-term remission by eliciting antitumor immune responses before primary tumor resection (34). This therapy correlates with improved pathological responses, enhanced disease-free survival, and expansion of T and B cell reservoirs within the tumor. Additionally, compared to adjuvant therapy, neoadjuvant immunotherapy demonstrates higher efficacy in eradicating metastatic disease (35, 36). Marron, T.U. et al. (25)found that patients with tumor necrosis rates exceeding 50% after cemiplimab treatment exhibited stronger immune infiltration compared to patients with minimal or absent necrosis in surgical samples. Similarly, Kaseb, A.O. et al. (22), analyzing tissue samples pre and post-resection, correlated treatment response with immune cell infiltration. They discovered a correlation between T cell activation positive (VISTA+) bone marrow cell clusters and significant pathology reactions. This phenomenon aligns with other studies in various tumor types (37, 38). Xia, Y. et al. (21) further reported that compared to patients with large tumor lesions, neoadjuvant immunotherapy has shown promising outcomes, particularly in HCC patients, especially those with a single lesion. This may be attributed to the increased presence of immune cell infiltration and upregulation of immune pathways in smaller tumor lesions. Variations in individual responses to immunotherapy among different patients suggest a correlation between the immune status of tumor lesions and individual anti-tumor responses and survival benefits.

Regarding the safety of neoadjuvant immunotherapy, the overall odds ratio for grade 3-4 TRAEs was 0.35 (95% CI 0.24-0.51). Most immunotherapy-related adverse effects were manageable, some resolving after treatment discontinuation, and others responding well to corticosteroid therapy. Overall, neoadjuvant immunotherapy in HCC demonstrated safety akin to previous studies in gastrointestinal tumors (39).Furthermore, the highest occurrence rate of grade 3-4 adverse events reported in this study was 43% (reported by Kaseb, A.O. et al. (22)—the only RCT in this article). The study also found that the combination of nivolumab and ipilimumab led to a higher rate of grade 3-4 treatment-related adverse events compared to nivolumab alone. However, this difference, at 20% ([95% CI –14.7% to 38.7], p=0.69), was not significant, suggesting that different combination therapies might result in varied outcomes, prompting considerations on optimizing drug usage to maximize patient benefit. Furthermore, the overall surgical resection rate after neoadjuvant immunotherapy showed an OR of 3.08 (95% CI 1.66-5.72). Although most patients underwent surgery as scheduled, a small proportion might lose their surgical eligibility due to disease progression, potentially facing toxic effects. Therefore, comprehensive preparation for unforeseen risks during the perioperative period is necessary to maximize patient benefits and ensure treatment safety.

The results of subgroup analysis indicate that there is no significant difference in efficacy among the three immune checkpoint inhibitors (ICIs) in monotherapy. However, regarding different immune combination therapies, the efficacy of dual ICI treatment is superior to monotherapy, while monotherapy is better than immune combination targeted therapy. Conversely, adverse event occurrences present a contrasting scenario where the rate of Grade 3-4 treatment-related adverse events (TRAEs) is lowest in immune combination targeted therapy. Both targeted and immune therapies have gained wide clinical application in HCC, but single therapy may lead to resistance and offer limited clinical benefits. Studies suggest that their combination yields better treatment outcomes. For instance, the IMbrave150 trial (40) demonstrated significant improvements in 1-year survival rates (67.2%) and mPFS (6.8 months) in previously untreated advanced HCC patients receiving atezolizumab combined with bevacizumab, approved by the FDA and CSCO for first-line treatment in advanced HCC. Other combinations such as lenvatinib plus pembrolizumab, sintilimab plus bevacizumab, and camrelizumab plus apatinib have also shown promising results (41–44). Additionally, the anticipated IMbrave050 phase III clinical study released mid-term analysis results at the 2023 AACR conference, indicating a 28% significant reduction in the risk of recurrence, distant metastasis, or death in HCC patients post-radical treatment (surgical resection or ablation) with the immune+anti-angiogenesis combination therapy (T+A regimen) (45), demonstrating clear survival benefits. Mechanistically, studies found that immune-modulating drugs restored the immune-supportive microenvironment, while anti-VEGF drugs like bevacizumab improved immune suppression and aided in restoring vascular normalization for efficient drug delivery, allowing for lower doses of ICI to reduce adverse reactions (46–49). In our subgroup analysis, while the combination of targeted therapy and Immunotherapy did not show a significant superiority, with the short follow-up, long-term survival benefits post-surgery need attention, providing clinical evidence for selecting optimal treatment strategies in the future.

Furthermore, with the widespread application of immunotherapy in HCC, many researchers have observed that patients showing favorable responses can gain long-term survival benefits (50, 51). However, there remain numerous patients who do not respond to immunotherapy, and identifying “high-quality” patients remains a pressing issue. Studies in various solid tumors have shown that the response rate to PD-L1/PD-1 inhibitor treatment correlates with overexpression of PD-L1 in patients’ tumor tissues, serving as a predictive biomarker for immunotherapy sensitivity (52–54). In studies specific to primary liver cancer, such as CheckMate040 and CheckMate459, patients with PD-L1 expression ≥1% and PD-L1 expression<1% exhibited significant differences in median overall survival time (OS) and objective response rates (ORR) (55, 56). However, the KEYNOTE224 study showed that tumor cell PD-L1 expression levels were not associated with treatment response rates (57). Other biomarkers such as gut microbiota, circulating tumor DNA, tumor-infiltrating lymphocytes, among others, have also undergone extensive research in HCC (56, 58–61). However, most biomarkers lack validation from large prospective studies, and their specificity and sensitivity remain unclear. Within this paper, only three articles reported results related to immune biomarkers. Xia, Y. and Ho, W. J (19, 21). discovered that tumor-infiltrating B cells or high expression of DCs serve as immune biomarkers for anti-tumor immunotherapy. Additionally, they found that higher levels of DCs after neoadjuvant treatment corresponded to a lower likelihood of patient recurrence. D’Alessio, A (28). focused on endpoints, suggesting that immune cell infiltration, peripheral cell-free DNA (cfDNA), and gut microbiota composition could serve as predictive factors for the efficacy of Nivo+Ipi anti-tumor therapy. Similar findings have been confirmed in tumors such as melanoma, colon cancer, lung cancer, and others (62–64). Presently, precise and efficient prognostic biomarkers in combination or establish multidimensional predictive models to select populations benefiting most from immune therapy, thus maximizing patients’ survival prospects.

Nevertheless, this systematic review has certain limitations. On one hand, there are discrepancies in the included literature regarding treatment regimens, patient inclusion criteria, pathological and radiological response criteria, leading to study heterogeneity. On the other hand, since more than half of the data originates from conference abstract articles, most of these do not report follow-up survival indicators such as Disease-Free Survival (DFS), Overall Survival (OS), etc. Consequently, this paper cannot analyze whether neoadjuvant treatment contributes to long-term benefits for patients. Moreover, there are only a few articles reporting on biomarkers for the efficacy of neoadjuvant immunotherapy, and no established patterns have emerged yet.

## Conclusions

In conclusion, research indicates that neoadjuvant therapy demonstrates both effectiveness and safety in resectable HCC. Regarding its efficacy, we observed that neoadjuvant immunotherapy results in pathological or radiological responses in certain patients, and these individuals seemingly experience better survival outcomes. Concerning its safety, the overall incidence of grade 3-4 treatment-related adverse events (TRAEs) with neoadjuvant immunotherapy is relatively low and does not significantly affect subsequent treatment plans. In summary, neoadjuvant immunotherapy offers benefits in the treatment of resectable liver cancer. However, substantial, high-quality trials in the future remain imperative to offer sturdy data support.

## Author contributions

CT: Investigation, Resources, Supervision, Validation, Visualization, Writing – original draft, Writing – review & editing. YY: Data curation, Funding acquisition, Software, Writing – original draft, Writing – review & editing. YW: Conceptualization, Project administration, Supervision, Writing – review & editing. LY: Conceptualization, Funding acquisition, Methodology, Writing – original draft. CY: Formal analysis, Investigation, Methodology, Writing – original draft. YT: Funding acquisition, Resources, Visualization, Writing – review & editing. GF: Funding acquisition, Resources, Visualization, Writing – review & editing. DZ: Funding acquisition, Resources, Visualization, Writing – review & editing. XW: Funding acquisition, Resources, Visualization, Writing – review & editing.
