# Supplementary material for: Neoadjuvant Immune Checkpoint Inhibitors in hepatocellular carcinoma: a meta-analysis and systematic review
Source: Front Immunol. 2024 Feb 19;15:1352873. doi: 10.3389/fimmu.2024.1352873 (PMC10909934; doi:10.3389/fimmu.2024.1352873)
Supplement: Supplementary file 1 [file DataSheet_1.zip › assessment of the literature quality.pdf]

Figure S.Meta-analysis of the Efficacy and Safety of Immune Checkpoint Inhibitors as Neoadjuvant Treatment for Hepatocellular carcinoma

**AuthorYear**	A	B	C	D	E	F	G	H	I	G	K	L	SCORE
Shi, Y.H. et al.,2021	2	2	2	2	1	2	2	0	-	-	-	-	13
Su, Y.et al.,2021	2	2	2	2	2	2	2	0	-	-	-	-	14
Ho, W.J.et al.,2021	2	2	2	2	2	2	2	0	-	-	-	-	14
Marron,T.U.et al.,2022	2	2	2	2	1	2	2	0	-	-	-	-	13
Xia, Y.et al.,2022	2	2	2	2	2	2	2	0	-	-	-	-	14
Kaseb, A.O.et al.,2022	2	2	2	2	2	2	2	0	2	2	2	2	22
Chen, S.et al.,2022	2	2	2	2	1	2	2	0	-	-	-	-	13
Bai, X. et al., 2022	2	2	2	2	2	2	2	0	-	-	-	-	14
Song,T.Q. et al., 2023	2	2	2	2	1	2	2	0	-	-	-	-	13
D’Alessio,A. et al.,2023	2	2	2	2	2	2	2	0	-	-	-	-	14
Sun,H.C . et al.,2023	2	2	2	2	2	2	2	0	-	-	-	-	14

Note： A. A clearly stated aim； B. Inclusion of consecutive patients; C. Prospective collection of data ; D. Endpoints appropriate to the aim of the study; E. Unbiased assessment of the study endpoint; F. Follow-up period appropriate to the aim of the study; G. Loss to follow up less than 5%; H. Prospective calculation of the study size . The items are scored 0 (not reported), 1 (reported but inadequate) or 2 (reported and adequate);I. Appropriateness of Control Group Selection; G. Synchrony of Control Group; K. Comparability of Baseline Characteristics; I. Adequacy of Statistical Methods.

In the table, only the study by Kaseb, A.O. et al., 2022, is a randomized controlled trial, necessitating assessments for appropriateness of control group selection, synchrony of control group, comparability of baseline characteristics, and adequacy of statistical methods. The remaining eight studies are single-arm trials or case series studies, hence they do not require the aforementioned four assessments, and are marked with a hyphen (-).
